# Current News and Opinion

**Published:** 1882-05

**Authors:** 


### CURRENT NEWS AND OPINION.

CASES OF TRICHINOSIS have been discovered in Brooklyn and Hoboken.

THE PENNSYLVANIA STATE MEDICAL SOCIETY met at Titusville on May 10th.

THE AMERICAN MEDICAL ASSOCIATION will meet in St. Paul, Minn., June 6–9 inclusive.

A NURSES' DIRECTORY has been established by the College of Physicians of Philadelphia.

THE *Medical Record* says: "We have plenty of new drugs, but new remedies are pitifully scarce."

THE TRUSTEES of Harvard College have decided not to permit women to matriculate as medical students.

A NIGHT MEDICAL SERVICE is to be established in Washington, according to the Chicago *Medical Review.*

ZANDER gives hydrochloric acid instead of iron in all cases of chlorosis, with eminently satisfactory results.

DR. WIRT JOHNSON has been elected President of the Mississippi State Medical Association for the ensuing year.

CHARLES DARWIN, the distinguished naturalist, died on the twentieth of April. He was interred in Westminster Abbey.

MISS SARAH BURR, who died March 1st, has given nearly half a million dollars to the various medical charities of this city.

DR. JAMES R. WOOD, Professor of Surgery in Bellevue Hospital Medical College, died in this city, May 5th, aged 66 years.

THE MEDICAL AND CHIRURGICAL FACULTY of Maryland has decided to establish a Directory for nurses in the city of Baltimore.

OIL OF THYME, thymol, tannin, tonqua bean, and balsam of Peru are all recommended as modifying the disagreeable odor of iodoform.

THE *Medical Bulletin* for April is even ahead of its usual enterprise by giving a full-page portrait of the late Prof. Jos. Pancoast.

SIR WILLIAM JENNER has been unanimously re-elected President of the Royal College of Physicians for the ensuing year.

DR. C. B. WHITE, of New Orleans, one of the most distinguished practical sanitarians in the country, died in that city April 16th, aged 56 years.

Small-pox seems to be generally on the increase. A number of cases were recently imported on one of the steamships from Bremen, and developed several days after landing.

Dr. John T. Hodgen, one of the most prominent surgeons in the Southwest, and president of the American Medical Association last year, died in St. Louis, on April 29th, aged 58 years.

Sir John Rose Cormack, a distinguished English physician, and one of the translators of Trousseau's Clinical Lectures for the New Sydenham Society died recently, aged 67 years.

Dr. P. S. Jenkins reports two cases (*Med. Herald*) of acute arsenical poisoning from eating sardines. The sardines contained a large quantity of arsenic.

The Cincinnati Obstetrical Society has a membership of seventeen, two of whom are ladies. The proceedings of the society rank in value with those of the larger Eastern cities.

Dr. H. Knapp does not believe in the antiseptic method in ocular surgery. He believes his results during the last two years, without antiseptics, fully as good as those of others who used antiseptics.

Yankee enterprise may hide its head in humiliation. The English undertakers, according to the *Medical Record,* send around circulars to the medical men offering a percentage on the business sent them!

Five deaths following nerve-stretching for locomotor ataxia have been reported. The symptoms (vomiting, singultus, paralysis of the bladder, etc.) seemed to indicate serious injury of the cord or medulla.

A number of cases of general dropsy with albuminuria, occurring in the later months of pregnancy, are reported by M. Chantreuil, which were relieved and convulsions possibly prevented by an exclusive milk diet.

One Joseph Dobson, M.D., of Fairfield, Conn., has a violent anti-vaccination screed in the May number of the *Medical Tribune.* We commend him to the attention of his neighbor, Wile, of the *N. E. Medical Monthly.*

Ignipuncture of the cervix is the latest treatment announced for chronic metritis. The punctures are made with the thermo-cautery parallel to the axis of the cervical canal, and between one fifth and one inch in depth.

DR. JOHN P. GRAY, Superintendent of the Utica Asylum, was shot in the cheek, March 16, 1882, by a lunatic, the latter being under the delusion that he was divinely commissioned to avenge Guiteau. The wound is painful but not serious.

THE *Pacific Medical and Surgical Journal* is authority for the statement that in Providence, R. I., with a population of 104,000, not a single death from small-pox had occurred since 1875. The reason given is "general and careful vaccination."

DR. CHAS. F. BEVAN reports (*Md. Med. Journ.*, May 1, 1882) eight successful cases of excision of the initial lesion of syphilis. The history of the cases seems to leave no room for doubt that they were cases of true chancre.

DR. R. B. WINDER has been elected Dean of the Baltimore College of Dental Surgery, in place of Dr. Gorgas, who has resigned to accept a similar position in the Dental department of the University of Maryland.

THE VARIOUS OPERATIONS performed on Senator Hill, of Georgia, for epithelioma of the tongue, by Professor Gross, have not been followed by permanent success. Senator Hill is reported to be dying in consequence of an extension of the disease.

DR. WILSON G. REGESTER, for a number of years secretary of the Medical and Chirurgical Faculty of Maryland, and who held the position of State vaccine agent since its first establishment, died in Baltimore, April 22d, of heart disease. He was only thirty-seven years of age.

PROFESSOR SAML. D. GROSS, who has been Professor of Surgery in the Jefferson Medical College for twenty-six years, has resigned and been elected Professor Emeritus. The chair of surgery in the college has been divided between Dr. Samuel W. Gross and Dr. Jno. H. Brinton.

DR. L. P. YANDELL says: "It was declared a century ago by John Hunter 'that there are but three classes of skin diseases, one of which is cured by mercury and the iodides, a second by sulphur, and a third class which the devil himself can't cure.'" A knowing man was Hunter.

THE FIRST INSTANCE where physicians are mentioned in the Bible is 2 Chronicles 16: 12: "And Asa, in the thirty-ninth year of his reign, was diseased in his feet until the disease was exceeding great; yet in his disease he sought not the Lord, but to the physicians. *And Asa slept with his fathers!*"

We can no longer doubt. The identical micrococcus of gonorrhœa has been found in blennorrhœa neonatorum. He is present in variable numbers in the pus, his numerical proportions determine the benign or malignant nature of the process. So says Haab in the *Deutsche Med. Zeitung*.

Dr. J. J. Rockwell, of Winesburg, Ohio, reports in the *Ohio Medical Journal* the case of a woman who bore seven children in twenty-one months, five in less than one year. She gave birth to twins twice in succession and to triplets in a third labor. She was one of twins, as was also her mother. She died a short time after her last confinement.

And now it is reported that Koch has disproved the statement that the common hay bacillus can be transformed into the *Bacillus anthracis* by cultivation. This is rather a set-back to the *verwilderungs* theory from an unexpected quarter. The germ theory of disease seems to have a good deal of trouble in getting itself demonstrated.

To be copied into the practitioner's note-book : Inhalation of five to ten drops of amyl nitrite will break up the chill of malarial fever ; so will the hypodermatic injection of one sixth of a grain of muriate of pilocarpine. It is said that twenty drops of oil of turpentine will control the diarrhœa of typhoid fever. Two to five drops of wine of ipecacuanha three times a day will in the majority of cases check the vomiting of pregnancy.

Dr. E. P. Brewer, of Norwich, Conn., finds (*Med. Record*, May 6, 1882), as a result of a number of experiments, that skin grafts remain viable, *i.e.*, capable of growth on the surface upon which they are grafted, as late as thirty-six hours after being removed from their living connection. In one case the grafts removed from a cadaver seventeen hours after death were successfully implanted nineteen hours after removal from the dead body. These observations seem to us to need repetition and verification by others.

In the *Hahnemannian Monthly* for April, Dr. W. J. Martin, of Pittsburg reports one hundred and eighteen consecutive cases of typhoid fever treated in the course of twelve months. Of these 118 cases only *two* died. Such a very favorable record naturally attracts some attention. But the explanation of Dr. Martin's singular success is found perhaps in the following quotation from his report (italics ours) : "The recognition of typhoid fever is, as a rule, not a difficult matter, *but where any doubt exists, it is best to consider the case as typhoid until positive it is something else*" !

